# Botanical Description of British Plants

**Published:** 1805-08-01

**Authors:** 


### *Botanical Description of British Plants.*

( Continued from our last, pp. 62.—72. )

18. Colchicum. *C. autumnale. C. Anglicum purpureum et album.*

*Ang.* Meadow Saffron. Tube root. Naked lady.

*Gen. Desc.* Cal. 0. bloss. six-div. tube very long, extending down to the root. Capsule three, inflated, united.

*Spec. Desc.* *Leaves* flat, spear-shaped, upright. *Caps.* large, egg-shaped, with three very blunt angles. *Bloss.* pale purple, tube three-cornered; segment rather unequal; styles reaching down to the root. *Germen* at the root surrounded by rudiments of the future leaves. It flowers in September, dies down and lies within the root all winter; in spring produces the fruit above ground, and ripens it the following summer: this may account for the great length of the styles, and affords, in the structure, an admirable instance of the wise contrivance of Providence. *See Withering, l. c. Meadows, low or mountainous, in rich soil.*

*Use.* The recent root of this plant, though denied by some to possess any acrimony, yet has been proved by the experience of Baron Stoerck, and others, to be extremely acrid, so as, cut into slices, to irritate by its particles the nostrils, fauces and breast, and even to have benumbed for a time the fingers on which it was held: taken internally, a single grain produced violent effects; and instances are recorded, in which it has proved a fatal poison both to man and brute animals. Stoerck first used it as a medicine; and the oxymel which he prepared from it, he found both *expectorant* and *diuretic,* and in various hydropic disorders eminently successful: he digested 1 oz. of the recent root, sliced, in 1 lb. of vinegar, for forty-eight hours with a gentle heat; the vinegar being strained proved still acrid, constringed and irritated the fauces, and excited excited a slight cough; to obviate this, he mixed the vinegar with twice its weight of honey, and gently boiled it down to the consistence of honey, forming a grateful oxymel, which promoted a copious discharge of urine, without producing any inconvenience from its acrimony, though it moderately stimulated the fauces and absterged the mucus: he recommends at first 1 drachm of this to be given twice a day in any suitable vehicle, and gradually to increase the dose to 1 oz. or more in a day. It has been found very successful in Germany and France, but in England the squill is thought more efficacious as a diuretic, and still more as an expectorant.—*Woodville.* It seems in its virtues very much to resemble the squill, but less nauseous and less acrimonious, though more sedative. *Withering.* An oxymel prepared from the roots gathered in the beginning of the summer, and administered in the quantity of 6 drs. to a boy, and 1½ oz. to a man, by a drachm at a dose, three or four times in the day, has, in several instances, cured the *dropsy*; but in some it has failed.—*Lightfoot.*

HEXANDRIA. HEXAGYNIA.

19. ARISTOLOCHIA. *A. clematitis.* *A. tenuis.*

*Ang.* Climbing Birthwort.

*Gen. Desc.* Bloss. six petals, tongue-shaped, entire; stamens near the germen. Caps. six-celled, beneath.

*Spec. Desc.* *Leaves* heart-shaped, alternate, blunt, shining above, pale green, smooth and veiny underneath. *Flowers* crowded, axillary, six or more together, scored, yellowish green, often tinged with purple. Caps. egg-shaped, blunt, pendent. *Stem* two or three feet high, simple, scored, cylind. zigzag, smooth. *Woods, hedges.* BL. July, September.

*Use.* The root has a somewhat aromatic smell, and a warm bitterish taste. Dr. Cullen says, that the difference of quality is not considerable between the different species of the A. but this seems to be preferred by both the London and Edinburgh Colleges. They are all considerably bitter, with more acrimony than many other of the bitters commonly employed. Its name seems to have arisen from the supposition of its *emmenagogue* virtues; and in some cases of retention and *chlorosis,* as a warm stimulating medicine, he has foumd it of use; but in cases of suppression he has never found it of any; and the commendation of it by the ancients in promoting lochia, facilitating birth, &c. is very ill formed. It has long been commended as a cure cure for the *gout:* it makes a considerable part of the Portland powder, and has often been employed by itself in the same manner as that powder, to be taken every day for a length of time. M. M. ii. 83.

But it is the opinion both of Cullen and Worlhoff, that though it may prevent the recurrence of the gouty paroxysms, yet the long continued use of such medicines is extremely hurtful, and commonly brings on a general state of disease more fatal than the original distemper.—*Woodville.*

### Class VIII. Octandria.

### Octandria. Monogynia.

1. Epilobium. *E. angustifolium. Sysimachia Chamænerion, Onagra.*

*Ang.* Rosebag willow-herb.

*Gen. Desc.* Cal. four-leaved, deciduous. Pet. four. Caps. beneath, four-celled, very long; seeds many, downy at the top.

*Spec. Desc. Stamens leaning. Leaves* scattered, strap-spear-shaped, with a few small teeth. *Bloss.* irregular, rose-colour, or white: petals entire. *Woods, hedges.* Bl. June, August.

*Use.* An infusion of this plant has an intoxicating quality, and the Kamskatkadales brew from the pith a sort of ale; and from the ale they make vinegar. The suckers of the root are eatable; the down of the seeds, mixed with cotton or fur, has been manufactured into stockings and other articles of cloathing. Goats are extremely fond of it: cows and sheep eat it: horses and swine refuse it.—*Withering.*

2. Epilobium. *E. hirsutum. Sysmachia siliquosa hirsuta.*

*Ang.* Large flowered willow-herb. Great hairy willow-herb. Codlings and cream.

*Gen. Desc. As above.*

*Spec. Desc. Stam. upright. Bloss* regular. *Pet.* cloven. *Leaves* egg-spear-shaped, hairy, half embracing the stem. *Stem* cylindrical, branched, hairy. *Petals* inversely, heart-shaped, fine rose colour, the claws white, with white scores spreading upwards. *Filam.* white. *Moist ditches, river banks, &c.* Bl. July.

*Use.* Horses, sheep, and goats eat it: cows are not fond of it: swine refuse it. The top shoots have a singularly delicate and agreeable fragrance, but so transitory, that before they they are gathered five minutes, it is no longer percep-tible: it resembles scalded codlings, and from thence the plant derives one of its names.—*Withering.*

3. Acer. *A. pseudo-platanus. A. majus.*

*Ang.* Sycamore-tree. Sycamore Maple.

*Gen. Desc.* Male flowers intermixed. Cal. five-cleft. Bloss. five-pet. caps. two or three; one-seeded, ending in a leaf-like expansion.

*Spec. Desc.* *Leaves* five-lobed, blunt, unequally serrated. *Flowers* in compound, pendent bunches, yellow green. *Pet.* very like the calyx. *Woods, hedges.* Bl. May, June.

*Use.* If a hole be bored into the body of a tree, when the sap rises in the spring, it will discharge a considerable quantity of sweetish, watery liquor, which is used in making wine, and, if inspissated, affords a fine white sugar. The wood is soft and very white: it is much used by turners for making bowls, trenchers, &c. This tree flourishes best in open places and sandy ground, but thrives very well in richer soil: it grows quick, is easily transplanted, bears cropping, and does not injure the grass under its shade. But it is said to grow best near the sea, and that a plantation of sycamores at fifty feet asunder, with three *sea sallow thorns* between every two of them, will make a fence sufficient to protect the herbage from the spray of the sea. *Gent. Mag.* 1757, *p.* 252. The pollen appears globular in the microscope; but if touched with any thing moist, these globules burst open with four valves, which then appear in the form of a cross.—*Withering.* Maple wood is also much used for the lathe; the Acer campestre.

4. Vaccinium. *V. myrtillus.*

*Ang.* Black worts. Black whortle berries. Hurtle-berries. Bil-berries. Wind-berries. Blea-berries. Bilberry whortle.

*Gen. Desc.* Cal. sup. Bloss. one-pet. filaments fixed to the receptacle. Berry four-celled, many-seeded; beneath, dimpled.

*Spec. Desc.* *Stam.* 10. Fruit-stalk one-flowered. *Leaves* bluntly serrated, egg-shaped, alternate. *Stem* and *branches* four-cornered. *Bloss.* seg. five; nearly globular, flatted at the base, pale reddish purple, mouth very small, with five small reflected teeth, reddish white. *Caps.* cells five. *Berry* blue black. *Woods, heaths.* Bl. April, May.

*Use.* The berries are often introduced at our tables: they are very acceptable to children, either eaten by themselves, or with milk, or in tarts. Their juice stains

2 linen linen or paper of a purple colour. The Moor-game live upon them in autumn. Goats eat this plant; sheep are not fond of it; horses and cows refuse it.—*Withering.*

5. Vaccinium. *V. uliginosum.*

*Ang.* Great Bilberry bush, or whortle. Rasp-berries.

*Gen. Desc. As above.*

*Spec. Desc. Leaves* very entire, inversely egg-shaped, blunt, smooth; when young, fringed at the base; flat, a net work of veins underneath. *Stem* one foot high. *Bloss.* pink. *Berry* blue, four slight angles; pulp white. *Moist woods, heaths, &c.* Bl. April, May.

*Use.* The juice of the berry is said to be frequently used by vintners in France, for the purpose of colouring their white wines red. Children sometimes eat the berries, but taken in large quantities they occasion giddiness and a slight head-ach, especially when full grown and quite ripe. Horses, cows, sheep, and goats eat it; swine refuse it.—*Withering.*

6. Vaccinium. *V. vitis-idæa.*

*Ang.* Red whortle-berries.

*Gen. Desc. As above.*

*Spec. Desc. Leaves evergreen*; inversely egg-shaped, entire, edges rolled back, dotted underneath, deeply veined above. *Stem* cylindrical, obliquely ascending, a span high. *Bunches* terminating, nodding. *Bloss.* pale pink. *Filam.* white, woolly. *Anthers* red, two cells, each with a yellow tube at the point; *berry* red. *Floral leaves* and *cups* coloured. It is seldom found in blossom. *Dry heaths, woods, mountain tops.* Bl. March, April.

*Use.* The berries of this species are acid, and not very grateful, but they are eaten by the inhabitants of Lapland, and by the country people; and they are sent in large quantities from W. Bothnia to Stockholm, for the purpose of pickling.—*Linn.* They are also made into tarts, rob, jelly, &c. Goats eat the leaves; cows, horses, and sheep refuse them.—*Withering.*

7. Vaccinium. *V. oxycoccos.*

*Ang.* Cran-berries. Moss-berries. Moor-berries. Fen-berries. Marsh whorts. Marsh whortle berries. Corn berries.

*Gen. Desc. As above.*

*Spec. Desc. Leaves evergreen*, egg-shaped, entire, edges rolled back. *Fruit stalks* single or in pairs, one-flowered, red, semi-transparent. *Stem* thread-shaped, trailing, not hairy. Cal. smooth, fringed at the points, coloured. *Bloss.*

four four distinct petals, deep flesh colour. *Stam.* often 10; *anth.* two-celled, ending in a hair-like open tube. *Style* red, tubular. *Summit* an open hole. *Berry* purplish red. *Peaty bogs.*

*Use.* At Long Town, in Cumberland, these berries are sold by the country people to the value of 20 or 30*l.* every market day, for five or six weeks together.—*Lightfoot.* Made into tarts they are in general much esteemed, but their peculiar flavour renders them disagreeable to some people. If they be wiped clean, and then closely corked in dry bottles, they will keep for several years; or the bottles may be filled with water. The name of Cranberry, which is the most general, originated probably from the fruit-stalks being crooked at the top, and before the expansion of the blossom, resembling the neck and head of a crane.—*Withering.*

[ To be continued. ]

